# The patient as a prosumer of healthcare: insights from a bibliometric-interpretive review

**DOI:** 10.1108/JHOM-11-2021-0401

**Published:** 2022-04-05

**Authors:** Maria Vincenza Ciasullo, Weng Marc Lim, Mohammad Fakhar Manesh, Rocco Palumbo

**Affiliations:** Department of Management and Innovation Systems, University of Salerno , Salerno, Italy; School of Business, Law and Entrepreneurship, Swinburne University of Technology , Melbourne, Australia; Department of Management and Law, University of Rome Tor Vergata , Roma, Italy; Faculty of Business, Design and Arts, Swinburne University of Technology , Kuching, Malaysia; Department of Management, University of Isfahan , Isfahan, Iran

**Keywords:** Patient, Prosumer, Co-production, Co-creation, Innovation, Service, Value, Healthcare, SPAR-4-SLR, Bibliometric, Interpretive, Review

## Abstract

**Purpose:**

Healthcare policies around the globe are aimed at achieving patient-centeredness. The patient is understood as a prosumer of healthcare, wherein healthcare service co-production and value co-creation take center stage. The article endeavors to unpack the state of the literature on the innovations promoting the transition toward patient-centeredness, informing policy and management interventions fostering the reconceptualization of the patient as a prosumer of healthcare services.

**Design/methodology/approach:**

A hybrid review methodology consisting of a bibliometric-interpretive review following the Scientific Procedures and Rationales for Systematic Literature Reviews (SPAR-4-SLR) protocol is used. The bibliometric component enabled us to objectively map the extant scientific knowledge into research streams, whereas the interpretive component facilitated the critical analysis of research streams.

**Findings:**

Patient-centeredness relies on a bundle of innovations that are enacted through a cycle of patients' activation, empowerment, involvement and engagement, wherein the omission of any steps arrests the transition toward service co-production and value co-creation. Institutional, organizational and cognitive barriers should be overcome to boost the transition of patients from consumers to prosumers in a patient-centered model of healthcare.

**Originality/value:**

The article delivers the state of the art of the scientific literature in the field of innovations aimed at sustaining the transition toward patient-centeredness and provides some food for thoughts to scholars and practitioners who wish to push forward service co-production and value co-creation in healthcare.

## Introduction

A transition toward a patient-centered model of healthcare characterizes the evolution of healthcare service systems across the globe, stimulating healthcare service providers to reconsider the design and delivery of healthcare services through the perspective of the patient ( Castro *et al.* , 2016 ). The bio-medical perspective associated with the provider-based model of healthcare is focused on treating (i.e. curing) the illness and understands the patient as a sheer recipient (or consumer) of healthcare services ( Adinolfi *et al.* , 2016 ). Patient-centeredness challenges this view, conceiving the patient as an active agent in the delivery of healthcare services, which are designed according to a dyadic approach based on service co-production and value co-creation ( von Thiele Schwarz, 2016 ; Ciasullo *et al.* , 2018 ). Patient-centeredness emphasizes the interpersonal nature of healthcare and relies on the assumption that patients and providers should coalesce to address issues related to health promotion and risk prevention ( Vrangbaek, 2015 ).

Patient-centeredness is a multifaceted construct entailing “… an approach that seeks to explore patients' desires, preferences, values, and concerns with the aim of empowering them to make decisions that best fit their individual needs” ( Liberati, 2016 : p. 343). From this standpoint, patient-centeredness comprises different nuances. First, it requires patient *empowerment* , which is a dynamic process intended to enable people to actively partake in the delivery of healthcare through (1) the enhancement of health knowledge and skills; (2) the improvement of self-efficacy; and (3) the participation in decision-making processes ( Markwart *et al.* , 2020 ). In sum, empowering patients allows them to have a voice in steering the provision of healthcare.

However, a gap between the theory and practice of patient empowerment has been reported by scholars as the idea and intention to empower patients does not necessarily materialize and lead to actual patient empowerment ( Aujoulat *et al.* , 2007 ; Lim, 2021 ; Yap *et al.* , 2021 ). To fill this gap, a process of *activation* is needed, which engenders the patient's “…propensity to engage in adaptive health behavior that may, in turn, lead to improved patient outcomes” ( Skolasky *et al.* , 2008 : p. 784). Hence, patient *activation* is a requisite to *empowerment* , underpinning the capability to understand health issues and to cope with them. Nevertheless, *activation* would not be possible if people do not possess competencies to establish a co-creating exchange with healthcare professionals and are unwilling to partner with them ( Pekonen *et al.* , 2020 ). Implementing patient-centeredness also relies on patient *engagement* and *involvement* , which make patients and providers eager to coalesce or come together for the purpose of value co-creation ( Ciasullo *et al.* , 2017 ). On the one hand, *engagement* entails “… a process in which patients, caregivers, and health professionals collaborate as equal partners, contributing unique skills while sharing information and perspectives toward innovative ideas” ( Rooke and Oudshoorn, 2020 : p. 497). On the other hand, *involvement* concerns the patients' propensity to adopt a participatory role in dealing with health-related issues ( Wensing and Baker, 2003 ). Engagement and involvement set the conditions for overcoming the provider-centered approach to healthcare – they put the patient at the center of healthcare provision and nurture healthcare service co-production, enriching patients' exchanges with healthcare professionals ( Palumbo, 2017 ).

Healthcare *service co-production* and *value co-creation* finalize the transition toward patient-centeredness. They reframe the patient as a consumer to a prosumer of healthcare, wherein the patient in the latter role acts as a resource integrator and value co-creator in collaboration with healthcare professionals as opposed to passively consuming prescribed healthcare services in the former role ( Framer *et al.* , 2017 ; Osei-Frimpong and Owusu-Frimpong, 2017 ). More importantly, a reconfiguration of organizational processes and management practices implemented by healthcare institutions is required for patients to enact the role of a prosumer. Failing to innovate extant structures and practices makes the transformation of the patient from a consumer to a prosumer of healthcare services unfeasible, impairing the transition toward patient-centeredness ( Palumbo and Manna, 2018 ).

Various attempts have been made to shed light on innovations conducive to an agile or a nimble transition toward patient-centeredness ( Palumbo, 2021 ). People-oriented strategies, structures and processes have been crafted to put people at the core of the healthcare system, soliciting the implementation of a participatory approach to healthcare ( Realpe and Wallace, 2010 ). Nevertheless, there is limited agreement on the *innovations* that can make patient-centeredness real ( Millenson *et al.* , 2013 ; Hansen *et al.* , 2022 ), which may be due to fragmented evidence. This calls for a systematization of extant scientific knowledge to find a synthesis among the various evidence reported in the scholarly literature and to achieve a holistic view of the path toward patient-centeredness ( Palumbo *et al.* , 2017a , b ; Proctor *et al.* , 2021 ). Existing reviews on this topic have addressed the *dimensions* ( Scholl *et al.* , 2014 ), *models* ( Zheng *et al.* , 2018 ) and *scales* ( Ree *et al.* , 2019 ) of patient-centeredness, some of which have been limited to specific niches of healthcare. The lack of systematization of scientific knowledge about the *innovations* enacting patients' *empowerment* and sustaining their *involvement* and *engagement* in the delivery of healthcare creates a knowledge gap, which prevents us from gaining a holistic understanding on the steps leading to patient-centeredness and thus inhibiting the transition of patients from consumers to prosumers of healthcare. Therefore, the review herein this article is guided by the following research question (RQ):

RQ. How do patient-centered innovations foster the transition of patients from consumers to prosumers of healthcare?

The article answers the RQ and fills the aforementioned knowledge gap by mapping and critically reviewing the scientific literature about the innovations that are expected to set the conditions for enabling patients to act as prosumers of healthcare. Noteworthily, a systematic review of the literature can provide valuable insights through a one-stop, state-of-the-art overview that future research can rely upon to advance the field ( Paul *et al.* , 2021 ). The article also contributes with an advancement of methods for literature reviews as independent studies, wherein both the contemporary bibliometric review and traditional interpretive review are integrated, supplementing quantitative techniques (e.g. bibliographic coupling) with human judgments, so that sensible interpretations are presented ( Donthu *et al.* , 2021 ).

The article begins with a disclosure of the review methodology, followed by the report of the review findings. Next, the critical discussion of the study results envisions avenues for further development. Lastly, the conclusion emphasizes its conceptual and practical takeaways, stressing the original contribution of this review.

## Methodology

Different types of systematic reviews are available to deliver a state of the art of the literature in a specific field, including domain-based, theory-based, method-based and meta-analytical reviews ( Paul and Criado, 2020 ). In line with the aim of this study, we undertook a domain-based review, which enabled us to obtain a comprehensive overview of scientific contributions related to a substantive research domain – i.e. innovation targeted to patient-centeredness. Domain-based reviews can take multiple forms, including bibliometric, conceptual, framework and thematic/interpretive reviews ( Paul and Criado, 2020 ). We adopted a *hybrid* approach, wherein *bibliometric* and *interpretive* analyses are jointly exploited to map the current literature and to review the steps that foster the reconceptualization of patients from consumers to prosumers of healthcare. The review relied on the *Scientific Procedures and Rationales for Systematic Literature Reviews* ( *SPAR-4-SLR* ) protocol by Paul *et al.* (2021) to *assemble* , *arrange* and *assess* relevant literature in the field.

### Assemble

The first step of the SPAR-4-SLR protocol is to *assemble* the materials for review, which involves *identifying* (i.e. review domain, research questions, source type and source quality) and *acquiring* (i.e. search mechanism and material acquisition, search period and search keywords) relevant scientific contributions.

We started identifying the *review domain* , which relates to patient-centeredness in healthcare. The *research question* pertained to innovations aimed at empowering patients to act as prosumers in the delivery of healthcare services. Considering its broad coverage and prestige ( Baas *et al.* , 2020 ), Elsevier's Scopus was queried as the main *source* of this review. Scientific contributions included for indexation in Scopus are typically academic in nature and have met the rigorous quality threshold for inclusion, such as peer review, publishing consistency and impact measures. Moreover, Scopus has been argued to represent a comprehensive and relevant source of information for conducting systematic reviews, which yields similar results to other available sources for conducting literature analysis, such as Clarivate Analytics' Web of Science ( Vieira and Gomes, 2009 ). Though Google Scholar has been argued to represent a valuable source for literature reviews ( Chertow *et al.* , 2021 ), it does not efficiently provide consistent and complete bibliometric data for reviews.

Next, we *acquired* scientific contributions. The *search mechanism* for material acquisition was targeted to the peculiar characteristics of the search engine embedded in Scopus. Since patient-centeredness is a well-established concept that attracted the attention of scholars and practitioners since the second half of the past century (e.g. Baden and Huebsch, 1971 ), we did not set a temporal limitation in terms of the starting publication year for this review in order to be as comprehensive as possible. Nonetheless, the *search period* was limited up 2020, as it is the most recent complete year at the time of writing, which is a search and review strategy that in line with Lim *et al.* (2022) . The *search keywords* relating to patient, innovation, and healthcare are accompanied by an asterisk to account for any potential variations of these terms – it was run in the “title, abstract and keywords,” as follows:

(TITLE-ABS-KEY (“patient eng*” OR “patient emp*” OR “patient inv*” OR “Patient act*” OR “patient enabl*” OR “patient co-cre*” OR “patient co-prod*”) AND TITLE-ABS-KEY (“innov*”) AND TITLE-ABS-KEY (“healthcare” OR “health care” OR “health org*” OR “health prof*” OR “healthcare prof*” OR “health care prof*”)) AND (EXCLUDE(PUBYEAR, 2021)) AND (LIMIT-TO(LANGUAGE, “English”))

The assembling stage was conducted on January 31, 2021 and yielded 533 articles. The bibliometric data and articles were collected in an electronic worksheet and a cloud drive, which were shared among the authors to perform arrangements in the next phase of the research protocol.

### Arrange

The second step of the SPAR-4-SLR protocol is to *arrange* the scientific contributions for review, which entails *organizing* (i.e. organizing codes) and *purifying* (i.e. defining exclusion and inclusion criteria) retrieved records. In terms of *organizing* , the bibliometric data is arranged using *codes* based on items' title, source, type, year of publication and citations. This enabled us to check the overall quality of retrieved contributions, which included articles published in peer-reviewed journals, proceedings of international scientific conferences, books and book chapters. At this stage, six duplicated items were found and removed from the dataset. In terms of *purifying* , the authors agreed on tailored *exclusion and inclusion criteria* to screen the available contributions. More specifically, the authors agreed to reject the records that (1) did not deal with patient-centeredness and/or with the reconceptualization of patients as prosumers of healthcare services (i.e. off-topic), (2) addressed topics related to the reconfiguration of patients as prosumers of healthcare services, but did not investigate innovation practices and processes to enacting such a transformation (i.e. off-scope), and (3) reported conceptual perspectives and/or critical commentaries on patient-centeredness, but did not significantly contribute to advancing what we know about the steps leading us toward the implementation of a patient-centered approach to healthcare (i.e. off-focus).

The authors independently analyzed the titles, abstracts and keywords of retrieved articles and excluded the articles that fell within one of the three above categories. At the end of the independent purifying activity, a meeting was held to achieve a consensus on those articles to be excluded from the analysis. The authors agreed on the exclusion of 253 articles, but there was disagreement on 48 articles. A debate ensued to discuss about the contested articles, wherein a majority rule was applied: if three of four authors agreed on the exclusion of the disputed article, then that article is removed from the dataset. The authors agreed on the exclusion of 34 of 48 articles under contention. In sum, 287 articles were removed from the dataset. More specifically, 78 articles were eliminated because they were off-topic, 82 articles were excluded because they were off-scope, and 127 articles were removed because they were off-focus.

### Assess

**Figure 1:**
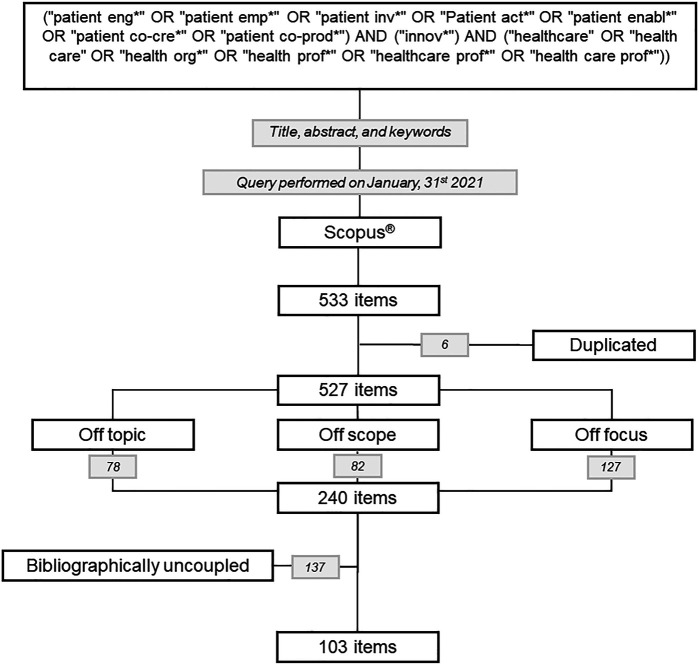
The flow-diagram depicting the scientific contributions' selection and analysis

The third and final step of the research protocol involved the *assessment* of selected items. On the one hand, the scientific contributions were *evaluated* through a bibliographic analysis to check their pertinence to a specific research stream. On the other hand, an interpretive approach was used to *report* the review's results. The bibliographic analysis was performed using VOSviewer (v.1.6.10), wherein the visualization of similarity techniques was exploited as the aggregation mechanism to systematize articles into clusters depicting homogeneous research streams ( Van Eck and Waltman, 2010 ). Bibliographic coupling, which was selected as the aggregation mechanism, is a bibliometric approach assuming that two articles citing one or more common references may belong to the same cluster ( Boyack and Klavans, 2010 ; Donthu *et al.* , 2021 ). VOSviewer performs this routine using a matrix based on the normalization of the co-occurrence of each article's references. The outcome of this routine is represented in a two-dimensional map, which locates items according to the similarity of their reference lists, wherein the nearer the articles, the stronger their relatedness. Bibliographic coupling was set at a minimum, allowing all selected articles to be included in the analysis. However, the total citation link strength was set at five, which is a decision made to enable a sharper focus on the core articles for each cluster. As a result, a total of 103 articles were found to be coupled into seven clusters. Figure 1 depicts a flow diagram summarizing the process of article collection and selection.

The clustered articles were carefully examined, using a follow-up *interpretive analysis* . The authors independently read the articles, trying to elicit the main themes that were addressed in each research stream. This is done because bibliographic coupling only provides quantitatively informed cluster groups without any explicit mention of the theme of the cluster representing the research stream. Specifically, an inductive coding approach was taken to delve into the clusters and obtain evidence of the main themes addressed across the seven research streams. The authors independently analyzed the articles. Next, they had a meeting to achieve a consensus on the naming and interpretation of the clusters. The gaps and tensions across the research streams were noted for future action as part of the *agenda proposal* for further developments.

## Findings

### Overview

**Figure 2:**
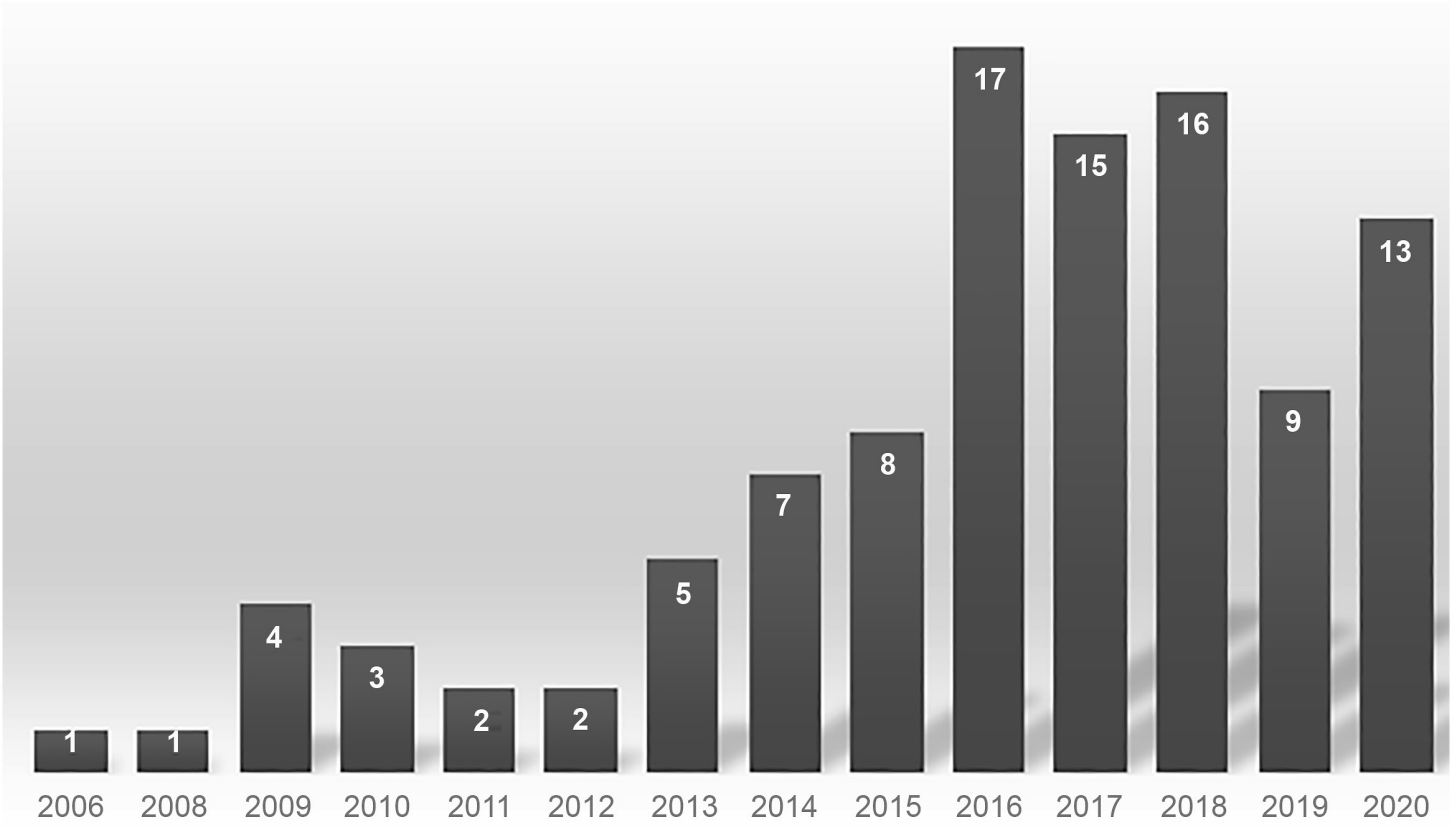
The distribution of selected items per publication year

The publication years of the body of literature focusing on innovations targeted to patient-centeredness ranged between 2006 and 2020. As depicted in Figure 2 , the investigation of patient-centered oriented innovations gained relevance in the past few years, spiraling post the new millennium. A steady growth of research endeavors is witnessed over the years, though a dip in 2019 and a recovery in 2020 were noted.

Most articles are published in peer-reviewed journals (70.9%). Review articles covered a sixth of the records (16.5%), though none of them overlapped in terms of content and purpose with this study. Four systematic reviews focused on specific topics, dealing with the conceptualization of healthcare co-production, the involvement of patients in health promotion and risk prevention initiatives, the establishment of online co-creation models for healthcare innovation, and the engagement of patients and informal caregivers in devising innovative healthcare provision models. Other reviews synthesized lessons learned from innovation processes addressed to patient-centeredness. Non-journal articles, such as book chapters (6.8%) and conference proceedings (3.9%), accounted for about one in 10 articles. An editorial and a research note were also included in this review.

Different subject areas were taken into consideration, including (1) medicine, (2) social sciences, (3) computer science, (4) business, management and accounting and (5) psychology. More than 70 different source titles were contemplated, with four peer-reviewed journals (i.e. *Patient Education and Counseling* , *BMC Health Services Research* , *Journal of Medical Internet Research* , and *Health Expectations* ) accounting for about one in five scientific contributions (19.5%). On average, the articles were cited 23 times, ranging from a minimum of 0 to a maximum of 277 citations. Articles published in peer-reviewed journals that had no citations at the time of writing were published in 2020.

### Thematic clusters

**Figure 3:**
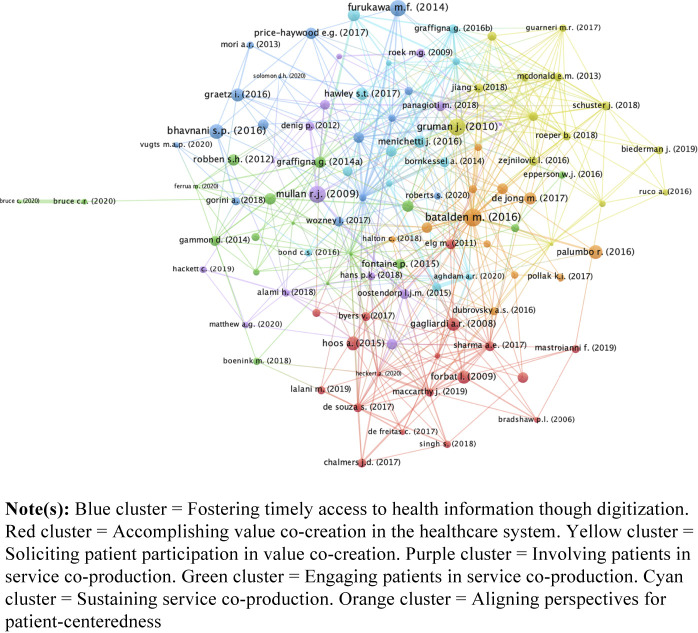
Thematic clusters obtained from bibliographic coupling

Figure 3 reports the results of bibliographic coupling. Altogether, seven clusters were identified, which focused on different research streams about innovations directed to fostering the conceptualization of patients as prosumers of patient-centered healthcare services. On average, the clusters consisted of 15 articles, ranging from a minimum of 12 articles to a maximum of 18 articles. The clusters received an average number of 300 citations, ranging from a minimum of 179 citations to a maximum of 476 citations.

An interpretive report of the clusters follows. As reported previously, a narrative approach was taken to summarize the main content of the different research streams embodied by the clusters. Although different research streams dealt with specific facets of management and organizational innovations addressed to accomplishing the shift of patients from consumers to prosumers of healthcare services, mutual connections can be identified across the clusters. This suggest that research endeavors intended to examine the role of patients as prosumers of healthcare services are merged by a common topic, which consists of the implementation of a patient-centered approach to healthcare.

#### Fostering timely access to health information though digitization (Blue cluster)

Enacting multiple gains for patients, such as an increased ability to keep track of health conditions and to obtain timely information about health-related issues and challenges, digitization is a cornerstone of the reconceptualization of patients as prosumers of healthcare services ( Bhavnani *et al.* , 2016 ). Digitization is key to activate patients, enabling the co-generation of personal health data and fostering the participation to healthcare decision making ( Aquino *et al.* , 2018 ). Moreover, digitization contributes to empowering patients, since it facilitates the exchange between patients and healthcare professionals and minimizes constraints produced by cognitive burdens, physical barriers and time limitations ( Mori *et al.* , 2015 ). Patients and healthcare professionals may exploit information and communication technologies (ICTs) and digital tools to monitor healthcare service quality and to assess the appropriateness of care, being able to reconfigure it to achieve better health outcomes ( Careyva *et al.* , 2015 ).

The design and the implementation of electronic health records (EHRs) has been identified as the starting point to digitize healthcare. As argued by Wass *et al.* (2017 : p. 211), “patients' online access to EHRs seems to be a step towards changing the role of the patient by enabling access to and providing patients with information that has previously been (…) less accessible.” The digitization of health records has twofold implications. On the one hand, it makes people able to autonomously obtain, process, and use information about their health, which is essential for their involvement in value co-creation ( Baudendistel *et al.* , 2015 ). On the other hand, it builds a bridge that fills the gaps between patients and providers, fostering knowledge sharing for health promotion and risk prevention ( Furukawa *et al.* , 2014 ).

The contribution of EHRs to patient-centeredness is especially salient when they are embedded in the ecosystem of digital tools and ICTs aimed at empowering patients and involving them in them provision of healthcare, such as web-based patient portals, telehealth applications, mobile health solutions and e-healthcare services. This makes it possible for people to record their experience with healthcare services and to use personal health data in a continuum of care perspective ( Greaves and Rozenblum, 2017 ). The pervasiveness of EHRs ensures the patients' access to adequate information to make effective health decisions, thus empowering them to have an active role in the design and delivery of healthcare ( Price-Haywood *et al.* , 2017 ). However, education and training activities should be delivered to patients to enhance their capability to handle health information stored in EHRs and to extract relevant cues from them ( Roberts *et al.* , 2020 ).

Literature has argued that an open approach should be adopted in designing EHRs, allowing people to contribute with self-reported data to increase the richness and the depth of health information available ( Solomon and Rudin, 2020 ). For this purpose, an alignment must be achieved between the functioning of EHRs and the patients' need for confidentiality and privacy ( Wozney *et al.* , 2017 ). Matching the effectiveness of EHRs and the privacy concerns of patients requires a strong collaboration among different stakeholders (e.g. policymakers, healthcare organizations, patients, informal caregivers and technology providers) involved in the design and implementation of EHRs ( Swinkels *et al.* , 2020 ).

Patients' access to timely health information is a requisite for the transition toward a patient-centered approach to healthcare. If an easy and comfortable access to information is missing, patients cannot get awareness of health conditions' determinants and their ability to learn from previous experiences is constrained, thus impairing their involvement as prosumers in healthcare service delivery ( Vugts *et al.* , 2020 ). Although the digital transformation sets the conditions for a patients' nimbler access to information about health-related issues, scholars have warned that people may be unwilling to use ICTs and digital tools to achieve a greater understanding of their health conditions ( Gorini *et al.* , 2018 ). From this standpoint, attention should be paid to the factors allowing patients to access health information in a friendly way, ensuring that the special needs of those categories of people who are less proficient in handling ICTs are addressed ( Graetz *et al.* , 2016 ).

#### Accomplishing value co-creation in the healthcare system (Red cluster)

Alongside enhancing the service relationship between patients and healthcare professionals through an improved exchange of data and information, digital technologies foster patient-centeredness sustaining a co-creation approach throughout the healthcare value chain, assigning to patients a guiding role in the functioning of the healthcare system ( Lalani *et al.* , 2019 ). Focusing on the design and delivery of healthcare, digitization allows patients to overcome the barriers hindering their active involvement in value co-creation and service co-production, such as inadequate physical infrastructures for patient engagement, difficulties in establishing direct relationships with providers, and hindrances in maintaining durable communication and information exchanges with them. Besides, looking at health governance, digitization empowers people to become prosumers by getting involved in shaping healthcare policies, priorities and research ( Maccarthy *et al.* , 2019 ).

Even though human-computer interactions may be exploited to advance the patient's ability to understand health issues and to actively participate in the functioning of the healthcare system, several precautions should be taken in developing digital solutions directed at patient empowerment. Patient empowerment and involvement are based on trust, which avoids that a lack of willingness toward engagement may arise due to the lack of human touch produced by the digitization of healthcare ( Blandford, 2019 ). From this standpoint, a co-design approach is needed in crafting human-computer interactions, encapsulating patients' concerns and perspectives in digital tools aimed at patient empowerment ( de Souza *et al.* , 2017 ). Moreover, the outcomes of patient empowerment should be continuously monitored to envision timely amendments to sustain the patients' participation in healthcare design and delivery ( Singh *et al.* , 2018 ).

Although they are necessary, digital resources are not sufficient in the recipe for value co-creation and service co-production. Patients' participation in value co-creation should be embedded in a networked approach to healthcare, according to which the providers of healthcare are encouraged to partner with patients to develop innovative healthcare delivery models that are consistent with patient-centeredness ( Brambini and Vang, 2018 ). The successful implementation of a networked approach to healthcare relies on several preconditions. A participatory model of healthcare enables patients to have a voice in inspiring the strategic decisions undertaken in the healthcare system. This requires embracing patients' advocacy as the founding value to enact a shift from a provider-based healthcare system toward patient-centeredness, wherein the patient evolves from a consumer to a prosumer of healthcare services ( Bradshaw, 2006 ). Next, parallel training activities set the conditions for patient-centeredness. On the one hand, patients should be provided with adequate knowledge and expertise to establish a fruitful partnership with healthcare professionals. On the other hand, healthcare professionals should acquire the social and communication skills that are required to engage patients in a co-creating dialogue ( Byers, 2017 ), dismantling the conventional biomedical approach to healthcare ( Gagliardi *et al.* , 2008 ). Finally, a customization of patient empowerment initiatives is required to align healthcare delivery models with the patients' health needs and expectations. The personalization of patient engagement allows healthcare professionals to comply with the ethical requirements of healthcare, minimizing the risk that the involvement of patients may undermine the integrity of healthcare delivery ( Sharma and Grumbach, 2017 ).

Drawing on these considerations, large-scale settings have not been argued as proper contexts to implement value co-creation initiatives, even though digital technologies enable a boundaryless approach to healthcare. Rather, value co-creation should be contextualized in small environments augmented by digital technologies to express its full potential, where healthcare professionals are more likely to understand the peculiar healthcare needs of patients ( Forbat *et al.* , 2009 ). Small-scale is conducive to better patient-to-patient exchanges, which further increase the effectiveness and the friendliness of patient empowerment, propelling a greater willingness of people to participate as prosumers in value co-creation ( Elg *et al.* , 2011 ).

#### Soliciting patient participation in value co-creation (Yellow cluster)

Different strategies pave the way for a reconceptualization of the functioning of the healthcare system in light of a patient-centered perspective ( Zejnilović *et al.* , 2016 ). In most cases, ICTs and digital tools have been exploited to increase the patients' functional adherence to medical prescriptions: text messaging and digital notifications are especially useful for this purpose, providing people with advice, aids, recommendations and updates to enhance the therapeutic adherence ( Biederman *et al.* , 2019 ). Such solutions can be embedded in more articulated digital architectures, such as apps for mobile devices and e-health tools, which are designed to ensure the access of patients to comprehensive support and assistance to effectively cope with health-related issues ( Jiang and Hong, 2018 ).

While they provide patients and informal caregivers with a functional assistance, these solutions fall short in delivering adequate social and emotional support, which is essential for the purpose of patients' activation and involvement in health protection and risk prevention ( Roeper *et al.* , 2018 ). Emotional and social support are the main scope of digital health communities, which rely on a thick network of web-based links to increase the patients' awareness of health challenges and to nurture their willingness to be actively involved as prosumers in value co-creation. Boosting the patient's ability to seek for adequate health-related information, to process available information to make timely decisions, and to navigate the healthcare system, digital communities foster the shift from patient education to patient engagement and, thus, from consumers to prosumers of healthcare ( Gruman *et al.* , 2010 ). Moreover, they solicit the patients' understanding of health issues and stimulate behavioral changes ( Guarneri *et al.* , 2016 ), with a positive contribution on health outcomes ( McDonald *et al.* , 2013 ). This is especially true when frail patients are concerned, including people suffering from mental health problems, who may greatly benefit from a behavioral approach to healthcare powered by digital technologies ( Schuster *et al.* , 2018 ).

Sticking to these considerations, scholars have argued that the design of a digital-based healthcare delivery system is established on four layers, which account for the cognitive, social, technological, and emotional determinants of patient empowerment ( Fico *et al.* , 2015 ). The first layer consists of databases storing information about health issues, diseases and health promotion and risk prevention initiatives. Alongside increasing the patients' access to relevant information, such tools allow healthcare professionals to monitor health treatments' outcomes and to recommend timely interventions to avoid the decline of health conditions ( Peek, 2010 ). The second layer is composed of patient platforms and portals, which are aimed at coaching patients to ensure their durable involvement as prosumers in a therapeutic alliance with healthcare professionals ( Ruco and Nichol, 2016 ). The third layer includes IT architectures aimed at creating a more direct link between patients and healthcare professionals to engage them in a co-creating effort for health promotion and risk prevention ( Jackson *et al.* , 2018 ). Lastly, social networking enacts peer-to-peer exchanges among patients, which further solicit their emotional involvement in value co-creation ( Lim, 2016 ).

#### Involving patients in service co-production (Purple cluster)

Patient-centeredness is rooted on the co-production of healthcare services ( Palumbo, 2016 ). Although healthcare professionals report contrasting assessments of the contribution of ICTs and digital tools in empowering patients for the purpose of healthcare service co-design and co-delivery, some evidence about the enabling role of such technologies have been highlighted in scientific literature ( Hans *et al.* , 2018 ). As previously anticipated, ICTs and digital tools enable secure and ubiquitous access of patients to personal data, enabling them to gain control over the information about their health, which is conducive to co-production ( Hackett *et al.* , 2019 ). Moreover, digitization empowers patients to act as co-creators of health information, which facilitates the establishment of a co-creating relationship with healthcare providers ( Teixeira and Suomi, 2011 ).

Digital technologies also permit patients to monitor the evolution of their health status and to collaborate with healthcare professionals to customize care and treatment considering the health outcomes that are achieved ( Fischer *et al.* , 2020 ). Sharing data about the evolution of the individual health condition prompts healthcare professionals and patients to engage in a lengthwise cooperative effort, which is intended to train the latter about the behaviors and the decisions that contribute to the improvement of their well-being ( Roek *et al.* , 2009 ). Such training fosters self-management of care to overcome risk factors that might trigger an exacerbation of health conditions ( Jacob and Serrano-Gil, 2010 ).

The combination of greater access to information and empowerment through coaching and training increases the patients and healthcare professionals' willingness to co-produce healthcare services, paving the way for their conjoined involvement in health promotion and risk prevention initiatives ( Matthew and Yang, 2020 ). For this to happen, several interventions are required, including the development of decision aids to support patients in self-managing their health conditions, the design of support materials to encourage patients to prioritize behaviors that maximize health outcomes, and the enhancement of interpersonal exchanges to engage patients in setting and achieving sustainable health-related goals in collaboration with healthcare professionals ( Denig *et al.* , 2012 ).

Although literature emphasizes the potential advantages that are disclosed by digital technologies to empower patients from the perspective of healthcare service co-production (e.g. Wickramasinghe and Gururajan, 2016 ), research has not found consistent evidence about the impacts of these interventions. Some factors may explain the ambiguous implications of initiatives intended to empower patients and to engage them in healthcare service co-production, such as (1) a limited proclivity of providers toward patient engagement, which makes it a rhetoric rather than a reality; (2) a scattered participation of patients in the co-design of solutions aimed at empowerment; and (3) the loss of human touch generated by the adoption of ICT-based solutions to involve patients ( Oostendorp *et al.* , 2015 ).

#### Engaging patients in service co-production (Green cluster)

Patients are more likely to engage with digital tools when no action is required, but they merely provide information and data about health issues ( Bruce *et al.* , 2020 ). Such information permits patients to obtain insights about their health condition, nurturing a greater awareness of health determinants, without necessarily implying the adoption of self-care behaviors ( Hudson *et al.* , 2020 ). An example comes from remote patient monitoring systems, which enable providers to assess the patients' health conditions and to provide feedback on how to increase health outcomes or to prevent illness' exacerbation ( Ferrua *et al.* , 2020 ). These considerations are consistent with the patients' preference for human contact and personal interactions as strategies to foster their involvement in value co-creation ( Obro *et al.* , 2021 ).

This calls for the implementation of innovative approaches to healthcare that exploit ICTs' pervasiveness and concomitantly rely on the human touch of traditional patient-provider interactions ( May *et al.* , 2018 ). Patient-centeredness, better patient-provider relationships and patient empowerment represent the pillars on which these innovative models of healthcare are established; however, little is known about their attributes ( Roark *et al.* , 2011 ). Despite this, scholars have emphasized their contribution to the enhancement of healthcare service quality, as well as to the reduction of costs due to an increased appropriateness of healthcare services ( Epperson *et al.* , 2016 ).

Several factors underpin the implementation of hybrid healthcare delivery models based on the pervasiveness of ICTs and the human touch of patient-provider exchanges. First, to ensure patient-centeredness, the evolving health needs and demands of patients must be accounted for throughout the different steps of healthcare service delivery, ranging from healthcare design to quality assessment ( Rubenstein *et al.* , 2014 ). Second, ICTs and digital tools should be integrated in healthcare delivery with a threefold purpose: (1) they should enable a continuous exchange among patients, informal caregivers and healthcare professionals ( Robben *et al.* , 2012 ); (2) they should coach patients, allowing them to fully recognize their healthcare needs and to be actively engaged in health promotion and risk prevention initiatives ( Graffigna *et al.* , 2014 ); and (3) they should entitle patients with a greater control over resources available for wellbeing improvement ( Gammon *et al.* , 2014 ). Third, attention should be paid to the whole patient experience, implementing a patient-centered approach to healthcare that minimizes grey areas undermining the comprehensiveness of care ( Cook *et al.* , 2015 ). Finally, yet importantly, an integrative change management approach should be undertaken, which jointly leverages the transformation of organizational cultures, the reconfiguration of healthcare delivery systems, and digitization to foster a transition toward personalized care, where engaged patients play an active role of healthcare service prosumers ( Fontaine *et al.* , 2015 ).

#### Accounting for the soft side of service co-production (Cyan cluster)

Patient-centered healthcare delivery models aimed at re-conceptualizing the patient as a prosumer of healthcare services include five main elements that are favorable for integrating conventional healthcare services with digitally enabled factors, namely (1) the extension of communication among patients, caregivers and healthcare professionals in a cyber-physical domain; (2) the improvement of data transparency allowing people to extract meaningful insights from health information; (3) the enhancement of individual and organizational health literacy, setting the conditions for a co-creating relationship between patients and healthcare professionals; (4) the design of support systems for patients and informal caregivers assisting them to navigate the healthcare system; and (5) patient empowerment ( Aghdam *et al.* , 2020 ). These elements confirm that digital technologies should be exploited to advance and enrich the patient-provider relationship, rather than substituting it with a high-tech, but low human touch experience ( Barello and Graffigna, 2018 ).

It is worth noting that patient-centeredness does not merely rely on the healthcare professionals' ability to design a technically consistent model of healthcare incorporating ICTs and digital platforms within conventional healthcare delivery models. Rather, it requires the acknowledgment of the special cognitive and behavioral needs of patients, which should be carefully contemplated in initiatives intended to promote people engagement in value co-creation. Inability to account for these soft factors leads to a deterioration of healthcare professionals' ability to involve patients as prosumers, making engagement a chimera, rather than a reality ( Palumbo *et al.* , 2017a , b ). Healthcare professionals should delve into the cultural and social factors influencing the patients' ability to take advantage of innovative healthcare delivery models and to cope with the cognitive and psychological barriers to patient empowerment: overlooking these issues may trigger a negative drift toward patients' disengagement ( Bond *et al.* , 2016 ).

These considerations are especially true for those categories of people who experience greater difficulties in getting empowered, such as those suffering from stigma related to their health conditions ( Wachira *et al.* , 2018 ) and underprivileged or underserved groups of the population, such as immigrants and cultural minorities ( Hawley and Morris, 2017 ). An ecological approach boosted by a personalization of healthcare services should be adopted to cope with the manifold factors that affect the empowerment of such categories of patients, addressing the contingent and socio-demographic variables that prevent people from getting a starring role as prosumers in the co-design and co-delivery of healthcare ( Graffigna *et al.* , 2013 ; Menichetti *et al.* , 2016 ).

#### Aligning perspectives for patient-centeredness (Orange cluster)

The viability of patient-centeredness depends on the empowerment of both patients and healthcare professionals, who should partner to evenly contribute to the improvement of health outcomes based on a co-creation and co-production approach ( Gill, 2013 ). The aims of the co-creating partnership between patients and healthcare professionals is twofold. On the one hand, it is oriented at aligning their perspectives about service provision, which is essential to achieve co-production. On the other hand, it should be intended to encourage the customization of healthcare service delivery, shaping it considering the patients' specific demands and needs ( Lau-Walker *et al.* , 2016 ).

Three organizational interventions are required to accomplish these two aims. First, a systemic approach to design comprehensive healthcare paths should be undertaken, enabling patients to be continuously engaged as prosumers in healthcare service co-production and overcoming gaps in the continuum of care that are prejudicial to patient engagement ( Vaccaro *et al.* , 2019 ). Second, healthcare professionals should be trained to involve patients as prosumers in value co-creation, increasing the individual and collective awareness of the gains that are heralded by service co-production and value co-creation ( Batalden *et al.* , 2016 ). Third, providers and users should be actively involved in the co-design of patient-centered healthcare delivery models, which should not be mandated by institutional policies and standards: this reduces resistances to change and facilitates the transition toward patient-centeredness ( Pollak *et al.* , 2017 ).

Such organizational interventions enable providers to wear the patients' shoes as prosumers and to understand the factors that determine the acceptability of patient engagement or, alternatively, discourage people from being involved in healthcare service co-production ( Foster *et al.* , 2016 ). Furthermore, they increase the healthcare professionals' commitment to empowering the patients, guiding them in a patient-centered healthcare system that conceives users as service co-producers, rather than as recipients of healthcare ( Halton and Cartwright, 2018 ).

## Discussion

### Insights and implications for theory and practice

**Figure 4:**
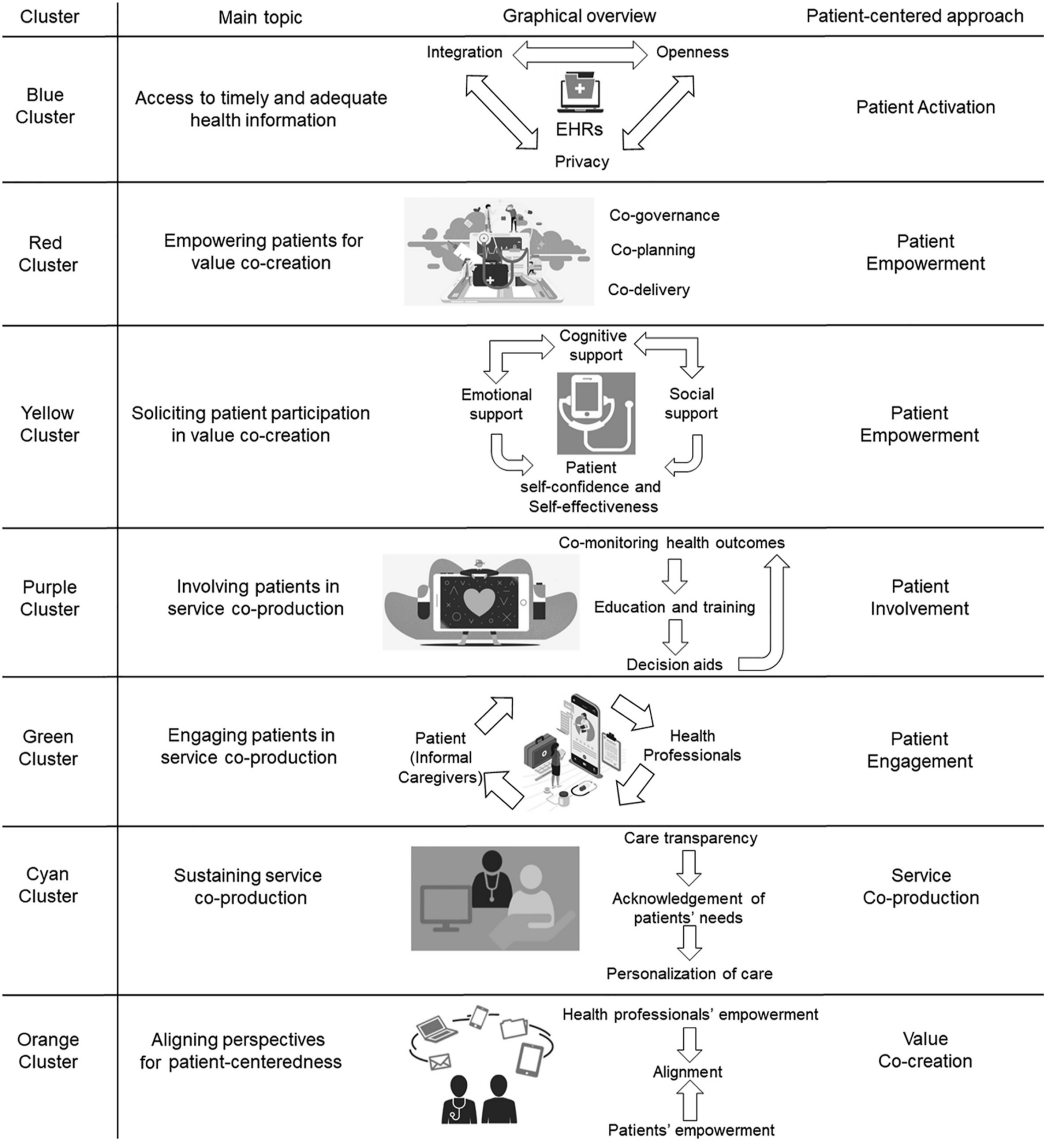
A graphical encapsulation of review findings

A graphical synthesis of the main insights that can be obtained from our review is presented in Figure 4 . The transition toward patient-centeredness in healthcare is established on a bundle of innovations, including both hard interventions on systems and architectures and soft actions on the approaches to healthcare enacting the transition of patients from consumers to prosumers of healthcare services. Embracing a patient-centered approach to healthcare implies that patients and informal caregivers play a starring role in the design and delivery of health promotion and risk prevention activities. Access to adequate and timely information is needed to empower patients and informal caregivers, enabling them to perform as value co-creators and service co-producers in partnership with healthcare professionals. From this point of view, arranging a reliable backbone to allow people to obtain, understand, process and use health information is a preliminary step in the transition toward patient-centeredness, as well as in the reconceptualization of patients as prosumers of healthcare services. EHRs are crucial to provide patients and healthcare professionals with a broader and timely access to health information, storing in a safe digital environment the whole health-related history of patients. The design of EHRs should be inspired by two principles. On the one hand, the functioning of EHRs is based on an open approach, which allows patients, informal caregivers and healthcare professionals to access health information and to contribute with the input of data about personal health conditions. On the other hand, integration is required to consolidate all information about the health history of patients into an interoperable repository. Obviously, openness and integration should be harmonized with the respect of patients' privacy, avoiding negative implications of EHRs on the confidentiality of health information.

Access to health information activates patients and makes them able to participate in the co-production of healthcare. Co-creation affects the different steps of the healthcare value chain, ranging from the governance of the healthcare system to the co-planning and co-delivery of health promotion and risk prevention services. Embedding digital technologies in the functioning of the healthcare systems enacts a process of patients' empowerment, which advances through patient advocacy and education and is established on the personalization of healthcare services. However, it is worth noting that digitization may negatively affect the patients' service experience, endangering their centrality in the delivery of healthcare. The dark side of digitization entails a loss of human touch in the design and the delivery of healthcare services, which impoverishes the patients' willingness to actively participate as prosumers in the value co-creation process.

Tailored solutions should be implemented to overcome the drawback of a high-tech, but low human touch healthcare delivery model on the patients' experience, ensuring the vividness of the patient-provider interaction and sustaining the patients' willingness to participate in the co-production of healthcare services. Web-based platforms and portals, digital communities, and social networks are especially relevant for this purpose, providing patients with the cognitive, social and emotional support they need to be engaged in value co-creation. Mobile health and e-health solutions foster a better exchange between patients and healthcare professionals, which is conducive to the establishment of a therapeutic alliance and to the enhancement of patients' ability to contribute as prosumers in the functioning of healthcare system. The digitization of healthcare facilitates the patients' participation in self-monitoring their health conditions and sharing data about health outcomes with healthcare professionals. Furthermore, it boosts the education of patients about health-related issues, training them to act as co-producers. Lastly, yet importantly, it supports health decision-making, providing patients and providers with aids to increase the appropriateness of healthcare in a perspective of patient-centeredness.

Despite these considerations, patients have been argued to prefer to use digital health solutions that do not require a direct action: people are willing to accept digitization of healthcare as soon as it does not require them to play an active role in the process of healthcare service design and delivery. This calls for critically discussing the effectiveness of digital health to accomplish a fully-fledged transition toward patient-centeredness. Digital solutions should be carefully integrated in conventional models of healthcare delivery, exploiting the potential of ICTs to build a durable exchange of information between patients and healthcare professionals. A hybrid healthcare delivery model, which jointly relies on the human touch of the traditional patient-provider interaction and on the pervasiveness of high-tech solutions, is required to boost patient empowerment and to enable patients to perform as value co-creators and service co-producers.

This hybrid healthcare model should be designed by considering the patients' cognitive and behavioral needs. An ecological approach should be embraced in configuring the functioning of the healthcare system, which recognizes the peculiar health needs of patients and exploits the flexibility of ICTs and digital tools to arrange a customization of health promotion and risk prevention services. Since this involves changing the conventional processes and practices implemented by healthcare organizations, attention should be paid to the empowerment of healthcare professionals, who should be trained and coached to undertake the perspective of patients as prosumers in co-planning, co-designing and co-delivering healthcare services. From this point of view, patient-centeredness requires an alignment of perspectives between patients and healthcare professionals, who should overcome their traditional conceptualization of users and providers to shift toward a co-creating relationship, wherein patients are empowered to partake in healthcare service co-production.

### Avenues for future research through the lens of theory, context and method

The takeaways obtained from our review inspire avenues for future research, which should be aimed at advancing our understanding of the hard and soft innovation required to foster the transition toward patient-centeredness. Three main streams for future research can be envisioned, which relate to the “theories,” “contexts” and “methods” of patient-centeredness for the prosumption of healthcare. Articulating future research directions focusing on theories (T), contexts (C) and methods (M) is in line with the TCM framework by Paul *et al.* (2017) : the trifecta of conceptual, empirical and methodological contributions is required to advance further research streams and to provide evocative insights about the peculiarities and requisites for engaging patients as prosumers of healthcare.

#### Theorizing patients as prosumers of healthcare

The reconceptualization of patients as consumers to prosumers of healthcare is based on their empowerment and engagement in healthcare service co-design and co-delivery. Although the literature is aware of the complexity lying behind the involvement of patients, there is a limited understanding of the intertwined institutional, organizational and management actions that should be undertaken to foster the shift toward a patient-centered approach to healthcare. Further theoretical advancements are strongly needed to untangle the multifaceted challenges that undermine patient-centeredness and obstruct the enablement of people as co-producers of healthcare services and value co-creators.

Value co-creation and service co-production are increasingly used as keywords emphasizing the integration of different perspectives framing the design of a patient-centered healthcare. On the one hand, value co-creation stresses the role of patients in co-planning, co-implementing, co-evaluating and improving healthcare services: it instils the mindset of patients' assumptions and believes among healthcare professionals, which should help to achieve patient-centeredness by aligning the perspectives held by users and providers. On the other hand, service co-production encapsulates the patients' active participation in the co-design and co-delivery of health promotion and risk prevention services, thereby recognizing them as partners – rather than customers – of healthcare professionals.

Drawing on these considerations, value co-creation and service co-production should be conceived as higher order constructs that need to be closely aligned with a service-centered view to shape a fully integrated patient-centered ecosystems ( Vargo and Lush, 2016 ). For this purpose, a set of lower order elements need to be accomplished. Service co-production and value co-creation rely on patients' activation, empowerment, engagement and involvement, which pave the way for a virtuous spiral leading to the establishment of a durable partnership among patients, informal caregivers and healthcare professionals. However, there is limited agreement about the conceptual building blocks of value co-creation and service co-production in healthcare. Improving the conceptualization of these two theoretical artifacts is expected to boost our ability to provide a fully-fledged understanding of patient-centeredness and to identify the steps forward to frame patients as prosumers of healthcare services.

#### Contextualizing the prosumption of healthcare

Literature suggests that healthcare service prosumption can be implemented in a myriad of contexts. In most cases, a hybrid approach to healthcare based on a balanced socio-technical infrastructure is required to provide patients with the information, knowledge and skills to actively participate in service co-production and value co-creation. To the best of the authors' knowledge, there is limited evidence of the ingredients that are needed in the recipe for establishing an empowering cyber-physical context. A balance between innovative healthcare delivery models powered by ICTs and the conventional patient-provider encounter is required to enable prosumption and to engage people in a co-creating partnership with healthcare professionals.

Future research should be aimed at examining the challenges that derive from the recontextualization of healthcare in a hybrid, virtual and physical domain. Particular attention should be paid to the backlash of virtualization on patients' involvement in the design and delivery of healthcare (e.g. the detachment of direct contact between patients and providers of healthcare services). By creating an immaterial wall between patients and healthcare providers, which consists of the digital devices used to access healthcare services, virtualization may entail the introduction of a cyber-biomedical approach to healthcare, which is dominated by healthcare providers and leave only limited space for patient empowerment.

These side effects of digitization should be clearly accounted for in future research, maintaining the need for a high-touch care to foster the transition toward patient-centeredness and to boost the reconceptualization of patients as prosumers of healthcare services. In this respect, future research adopting a psycho-socio-technical perspective are encouraged to inspire the arrangement and contextualization of a patient-centered healthcare system. It should unpack the contextual peculiarities of service exchanges among patients, informal caregivers and healthcare professionals, where digitization is fully integrated with conventional delivery models.

#### Methods for enabling the prosumption of healthcare

The implementation of prosumption requires a combination of hard and soft actions, which affect all the actors participating to the functioning of the healthcare system. Future research should illuminate the methods and the implications of hard and soft actions targeted to patient-centeredness, emphasizing their contribution to the empowerment of patients. Hard actions are directed at providing people with the instruments and with the skills that are needed to effectively participate in service co-production and value co-creation. Prospective studies should be aimed at unravelling the methods that are more effective in enhancing the patients' health literacy (i.e. their ability to navigate the healthcare service system), setting the conditions for their reconceptualization as prosumers of healthcare. Soft actions are directed to foster the patients' willingness to be involved as prosumers in the design and delivery of healthcare. This involves a change in the healthcare professionals' mindset, who should acknowledge patients as partners, rather than as mere recipients of healthcare. Actions for enhancing organizational health literacy (i.e. the organizational capability to empower people and to engage them in value co-creation) are especially relevant for this purpose, soliciting healthcare organizations to act as a co-creating environment, where patients, informal caregivers and healthcare professionals collaborate to address health issues and to achieve a sustainable well-being. Both quantitative and qualitative studies are required to collect empirical evidence on the role of health literacy and organizational health literacy in empowering patients, so that innovative healthcare delivery models, methods and practices can be arranged to enable patients to effectively perform as prosumers of healthcare services.

## Conclusion

Both conceptual and practical implications can be obtained from the results of this review. Patient-centeredness implies a reconfiguration of healthcare delivery models framing patients as prosumers in the design and delivery of healthcare. Achieving patient-centeredness relies on a virtuous and self-nourishing process of patients' enablement, which advances through four steps. First, an *activation* stage is realized, whereby patients acquire the capability to collect health information that are necessary to obtain an increased awareness of their health conditions and to recognize the resources available for health promotion and risk prevention. Next, an *empowerment* stage occurs, whereby patients gain the knowledge, skills and expertise to participate as co-producers in the provision of healthcare services. Third, an *involvement* stage enacts a durable dialogue between patients and healthcare professionals, in a perspective of continuous value co-creation. Fourth, an *engagement* stage entails a fully-fledged prosumption of healthcare services based on a therapeutic alliance between patients and healthcare professionals.

A bundle of innovations paves the way for accomplishing the transition toward patient-centered healthcare. Digital innovations intended to recontextualize the delivery of health promotion and risk prevention services in a digital environment are crucial to empower people, facilitating their access to timely health information and streamlining their interactions with healthcare professionals. Furthermore, innovative practices based on the durable exchange between patients and healthcare professionals are required to sustain the involvement and the engagement of people as co-producers of healthcare services. The recontextualization of the patient-provider relationships in a value co-creation perspective can help overcome the cognitive, emotional and social barriers that prevent patient engagement, promoting the transition toward patient-centeredness. Future innovations targeted at patient-centeredness should be established on a hybrid model, which concomitantly exploits the potential of ICTs and digital tools to empower patients. Alongside ensuring a durable, direct and friendly exchange between patients and providers, this minimizes the risk that the transition toward a digital-based healthcare delivery system disrupts the human touch of healthcare service provision, which is a key element for patient-centeredness.
